# Marine-derived EGFR inhibitors: novel compounds targeting breast cancer growth and drug resistance

**DOI:** 10.3389/fphar.2024.1396605

**Published:** 2024-05-01

**Authors:** Qi Li, Bo Li, Qian Wang, Chengen Wang, Miao Yu, Tianfu Xu

**Affiliations:** ^1^ State Key Laboratory of Natural and Biomimetic Drugs, School of Pharmaceutical Sciences, Peking University, Beijing, China; ^2^ Department of Minimally Invasive Tumor Therapies Center, Beijing Hospital, National Center of Gerontology, Institute of Geriatric Medicine, Chinese Academy of Medical Sciences, Beijing, China; ^3^ Engineering Research Center for Medicine, Ministry of Education, Harbin University of Commerce, Harbin, China

**Keywords:** breast cancer (BC), EGFR inhibitors, marine-derived compounds, apoptosis induction, EGFR kinase selectivity

## Abstract

Breast cancer (BC) continues to be a major health challenge globally, ranking as the fifth leading cause of cancer mortality among women, despite advancements in cancer detection and treatment. In this study, we identified four novel compounds from marine organisms that effectively target and inhibit the Epidermal Growth Factor Receptor (EGFR), crucial for BC cell growth and proliferation. These compounds not only induced early apoptosis through Caspase-3 activation but also showed significant inhibitory effects on EGFR mutations associated with drug resistance (T790M, L858R, and L858R/T790M), demonstrating high EGFR kinase selectivity. Cell Thermal Shift Assay (CETSA) experiments indicated that Tandyukisin stabilizes EGFR in a concentration-dependent manner. Furthermore, binding competition assays using surface plasmon resonance technology revealed that Tandyukisin and Trichoharzin bound to distinct sites on EGFR and that their combined use enhanced apoptosis in BC cells. This discovery may pave the way for developing new marine-derived EGFR inhibitors, offering a promising avenue for innovative cancer treatment strategies and addressing EGFR-mediated drug resistance.

## 1 Introduction

Breast cancer, a prevalent malignancy among women, poses a significant threat to their health and well-being (Torre et al., 2015). Characterized by its high heterogeneity, breast cancer presents a challenge in drug selection and sensitivity due to the vast molecular diversity observed even among patients with similar pathological features (Shigematsu et al., 2018). This variability often results in markedly different prognoses (Sun et al., 2021). The current clinical approach to treating breast cancer involves a comprehensive strategy primarily centered on surgery, with adjunctive treatments including radiotherapy, chemotherapy, endocrine therapy, and targeted therapy. The advent of molecular targeted therapy has revolutionized breast cancer treatment by focusing on the signaling pathways pivotal to the cancer’s genesis and progression. This method offers treatment at the cellular and molecular levels, enabling the specific elimination of cancer cells while sparing healthy surrounding tissue (Walker et al., 2015). Such advancements have significantly enhanced the prognosis for breast cancer patients, marking a new era in the biological treatment of this disease.

Targeted therapies for breast cancer specifically focus on molecules crucial for the growth and survival of cancer cells. Among these, HER2-targeting agents such as trastuzumab (Herceptin), pertuzumab (Perjeta), ado-trastuzumab emtansine (Kadcyla), and lapatinib (Tykerb) have shown significant efficacy (Swain et al., 2023). Additionally, CDK4/6 inhibitors, including palbociclib (Ibrance), ribociclib (Kisqali), and abemaciclib (Verzenio) (Pandey et al., 2019), offer a strategic advantage when combined with hormone therapy for certain breast cancer subtypes.

The Epidermal Growth Factor Receptor (EGFR), also known as ErbB1 or HER1, serves as the foundational member of the ErbB/HER family of receptor tyrosine kinases (RTKs). This receptor plays a critical role in regulating developmental processes via several signaling pathways, including the phosphatidylinositol 3-kinase (PI3K)/Akt, Ras/Raf/MEK/ERK, and STAT3 pathways (Burgess, 2008). EGFR, upon ligand binding, is capable of forming either homodimers with itself or heterodimers with its family counterparts-ErbB2/HER2, ErbB3/HER3, and ErbB4/HER4. This dimerization triggers conformational changes that activate the kinase domain of the receptor (Zhang et al., 2006; Ferguson, 2008; Jura et al., 2009). Overactivation of EGFR signaling, whether through point mutations, intragenic deletions, or overexpression, has been conclusively linked to the pathogenesis of various diseases, underscoring its significance in cellular regulation and disease development (Normanno et al., 2006; Roskoski, 2014).

Research indicates that the EGFR plays a crucial role in the initiation, progression, and metastasis of primary breast cancer (Lakis et al., 2016). Notably, EGFR is overexpressed in a wide range of breast cancer cells, including in approximately 50% of triple-negative breast cancers (TNBC). TNBC is characterized by the absence of estrogen receptors, progesterone receptors, and HER2, which contributes to poor clinical outcomes and a scarcity of effective therapeutic options (Nielsen et al., 2004; Alvarez et al., 2010; Harbeck and Gnant, 2017). In TNBC, the overexpression of EGFR leads to the constant activation of the PI3K/AKT/mTOR signaling pathway (El Guerrab et al., 2020). The synergistic application of EGFR and mTOR inhibitors has been shown to significantly enhance the anti-tumor efficacy of these drugs. Furthermore, combinations such as the EGFR-targeting drug erlotinib with bevacizumab, and cetuximab with cisplatin, have demonstrated substantial benefits in extending the survival time of breast cancer patients (Symonds et al., 2019) and alleviating symptoms (Baselga et al., 2013), respectively. Additionally, a clinical study (NCT00066378) has revealed that EGFR tyrosine kinase inhibitors (TKIs) can postpone the development of endocrine resistance in breast cancer. Consequently, EGFR presents a promising target for the development of effective breast cancer treatments.

Currently, third-generation drugs targeting the EGFR are predominantly employed in the treatment of non-small cell lung cancer (NSCLC). At the initial stages of NSCLC treatment, EGFR TKIs demonstrate significant efficacy. First- and second-generation EGFR-TKIs, such as gefitinib and afatinib, have shown considerable success in managing advanced NSCLC in patients with activating EGFR mutations. However, resistance often develops due to EGFR T790M mutations (Recondo et al., 2018), which do not hinder the binding of TKIs to EGFR but rather enhance the affinity of EGFR for ATP (Yun et al., 2008). To address the challenge of resistance mediated by EGFR T790M mutations, third-generation EGFR-TKIs, exemplified by osimertinib, achieve irreversible inhibition of EGFR kinase activity. This is accomplished through the formation of a covalent bond with the C797 residue of the EGFR kinase domain (Zeng et al., 2022). Despite this advancement, patients undergoing treatment with third-generation EGFR-TKIs may eventually exhibit resistance mechanisms independent of EGFR, often several months post-treatment. Presently, fourth-generation EGFR-TKIs are under development and have not yet received clinical approval, indicating that significant research and development efforts remain necessary to overcome these emerging resistance pathways.

The combination of breast cancer-targeting drugs with EGFR-TKIs holds promising potential in significantly enhancing drug efficacy, underscoring the importance of discovering a newer generation of EGFR-TKIs for breast cancer treatment. However, first-generation EGFR-TKIs exhibit severe hepatotoxicity and skin toxicity, while second-generation EGFR-TKIs present even more pronounced adverse reactions. Although third-generation EGFR-TKIs boast reduced incidence of adverse effects compared to their predecessors, they still pose risks such as diarrhea, rash, dry skin, paronychia, QTc interval prolongation on electrocardiograms, and interstitial pneumonia. Consequently, there exists an urgent imperative to explore novel EGFR inhibitors characterized by structural diversity, enhanced safety profiles, and heightened efficacy.

In our study, we identified a novel EGFR inhibitor, characterized by a unique chemical structure derived from marine organisms. This inhibitor demonstrated the capacity to concurrently suppress the enzymatic activities of both the wild-type EGFR and its pathogenic mutants at nanomolar concentrations, exhibiting notable selectivity for tyrosine kinase inhibition. Cellular Thermal Shift Assay (CETSA) results affirmed the inhibitor’s effective intracellular engagement with EGFR. Tandyukisin demonstrated significant inhibitory activity against cancer cells expressing high levels of EGFR, while exhibiting negligible inhibitory effects on both low EGFR-expressing cancer cells and normal human breast cells. These findings suggested that Tandyukisin possessed enhanced selectivity for cancer cells and exhibited reduced cytotoxicity towards normal cells. Moreover, our findings from flow cytometry and Western Blot (WB) analyses indicated that this inhibitor induced Caspase-3 activation via the mitochondrial pathway, thereby facilitating early apoptosis in cancer cells. Importantly, synergistic effects were observed when this inhibitor was used in combination with other inhibitors targeting distinct regions of EGFR, namely, the extracellular ligand-binding domain and the intracellular kinase domain, leading to a significant enhancement in the suppression of breast cancer cell proliferation.

## 2 Materials and methods

### 2.1 Materials

The extracellular ligand-binding domain of EGFR, tagged with His, and the intracellular tyrosine kinase (TK) domain of EGFR, tagged with GST, were acquired from Sinobiological, China. Kinase assay systems for both wild-type EGFR and its mutants—T790M, L858R, and the dual L858R/T790M—were procured from Promega Corporation, USA. Cell lines including MCF-7, A549, A431, H1299, HL-60, and MCF-10A were obtained from the China Infrastructure Cell Line Resources.

### 2.2 Cell culture

The cell lines MCF-7 and A431 were cultured in Dulbecco’s Modified Eagle’s Medium (DMEM, Gibco, USA), while HL-60, H1299, and A549 cells were maintained in RPMI-1640 medium (Gibco, USA). Both mediums were enriched with 10% fetal bovine serum and 1% penicillin/streptomycin. The MCF-10A cell line was cultured in a 1:1 mixture of DMEM and F12 medium (Gibco), supplemented with 5% horse serum, 10 μg/mL insulin, 0.1 μg/mL cholera toxin, 0.5 μg/mL hydrocortisone, and 0.02 μg/mL epidermal growth factor (EGF). All cell cultures were incubated at 37°C in an atmosphere containing 5% CO2.

### 2.3 MTT assay

Cells in the exponential growth phase were seeded into 96-well plates and allowed to adhere overnight. Subsequently, they were treated in triplicate with various concentrations of the compounds (0, 1.56, 3.13, 6.25, 12.5, 25, 50, 100 µM), either with or without. After 24 h, 20 µL of Methyl-thiazolyldiphenyl-tetrazolium bromide (MTT, 5 mg/mL, Thermo Fisher Scientific) was added to each well and incubated for a minimum of 4 h. Following the incubation, the medium containing the compounds and MTT was removed from each well, and 200 µL of dimethyl sulfoxide (DMSO) was added. The plates were then shaken for 10 min at 37°C to dissolve the formazan crystals. The absorbance was measured using a microplate reader (BioTek Synergy 4) at a wavelength of 490 nm.

### 2.4 Cell cycle and early stage apoptosis analysis

After 24 h of treatment with various concentrations of compounds, MCF-7 cells were harvested and washed twice with phosphate-buffered saline (PBS). For cell cycle analysis, the cells were initially fixed with 70% ethanol at 4°C overnight and subsequently washed with PBS. The fixed cells were then resuspended in 1× PBS containing 0.5% Triton X-100, 50 μg/mL propidium iodide (PI), and 50 μg/mL DNase-free RNase A, and incubated at 37°C for 30 min in darkness. Following incubation, cell cycle distribution was assessed using a BD FACSCanto™ flow cytometer.

Apoptosis was evaluated using the Annexin V-FITC/PI Apoptosis Detection Kit (Vazyme). The harvested MCF-7 cells were first resuspended in 1× binding buffer from the kit to achieve a final concentration of 1×10^6^ cells/ml. Next, 100 μL of the cell suspension, 5 μL of Annexin V, and 10 μL of PI were mixed and incubated in the dark for 15 min. Subsequently, 400 μL of 1× binding buffer was added to the mixture in preparation for apoptosis analysis.

### 2.5 Western blotting

MCF-7 cell lysates were prepared by rinsing the cells twice with PBS and then solubilizing them in lysis buffer (NP40 supplemented with protease and phosphatase inhibitor cocktails) on ice. The solubilization process involved rotating the lysis buffer/cell mixture end over end for 10 min at 4°C. After centrifugation for 10 min at 4°C to remove insoluble material, the protein concentration of the solubilized protein was determined using a BCA assay. Subsequently, samples were diluted in SDS sample buffer. Equivalent amounts of protein were separated by SDS-PAGE, transferred to PVDF membranes, and probed with the indicated antibody. Prior to incubation with primary antibodies overnight at 4°C, the membranes were blocked with 5% skim milk-TBST. The antibodies and their sources were as follows: PARP1 (1:500, Santa Cruz Biotechnology), Cleaved Caspase-3 (1:1000, Cell Signaling Technology), BAX (1:500, Santa Cruz Biotechnology), BCL-2 (1:500, Santa Cruz Biotechnology), and GAPDH (1:500, Santa Cruz Biotechnology). Following incubation with horseradish peroxidase-conjugated goat anti-mouse or goat anti-rabbit secondary antibody (Santa Cruz Biotechnology), the detected proteins were visualized using the Pierce Enhanced Chemiluminescence (ECL) reagent and a UV Products imaging system. Band intensities were quantified using the National Institutes of Health ImageJ software.

### 2.6 Kinase inhibition assays

The experiments were conducted in accordance with the manufacturer’s guidelines (Promega). The procedure began with a kinase reaction, where 5 μL of 1× kinase buffer was utilized, and the mixture was incubated at room temperature for 60 min. This step was followed by the addition of 5 μL of ADP-Glo™ reagent to terminate the kinase reaction and to deplete any remaining ATP, with a subsequent incubation period of 40 min at room temperature. Next, 10 μL of kinase detection reagent was added to convert ADP back into ATP, and luciferase along with luciferin was introduced to facilitate ATP detection. This mixture was then incubated at room temperature for an additional 30 min. Detection was carried out using a BioTek Synergy4 instrument, and data analysis, including curve fitting and presentation, was performed using GraphPad Prism version 5.0. Staurosporine, a potent indolecarbazole alkaloid, was employed as a specific inhibitor of protein kinases to validate the specificity of the assay (Omura et al., 1977; Song et al., 2017; Abou-Zied et al., 2019).

### 2.7 EGFR thermal stability analysis

In the CETSA, MCF-7 cells were treated with varying concentrations of Tandyukisin (5, 10, 20 µM) or a control solution of 0.2% DMSO for 24 h. Following treatment, cells were harvested and washed with PBS to eliminate any residual drug, then an equal number of cells were allocated into 0.2 mL PCR tubes. These cells were resuspended in 100 µL PBS supplemented with a complete protease inhibitor cocktail and subjected to a heating step at 50°C for 3 min. Post-heating, the cells underwent lysis through three cycles of freeze-thaw using liquid nitrogen, followed by centrifugation at 12,000g for 20 min at 4°C to separate the lysate. The clarified supernatant from each tube was then carefully transferred to fresh tubes for Western blotting.

In the Differential Scanning Fluorimetry (DSF) study, concentrations of Tandyukisin-1.3, 2.5, 5, and 10 µM-were incubated with 0.05 mg/mL of the EGFR kinase domain. A control sample, containing only 5% DMSO, was used alongside the EGFR kinase domain. The thermal stability of the EGFR kinase domain was assessed using PR NT.48 (NanoTemper). The assay conditions were set to a temperature range of 25°C–95°C with a ramp rate of 1 °C/min.

### 2.8 Surface plasmon resonance (SPR) experiments

The binding affinities of compounds towards EGFR extracellular ligand binding domain and EGFR intracellular kinase domain were assayed using the SPR-based Biacore 8K instrument (Cytiva). Considering the purchased EGFR was fused with GST tag, GST alone was also immobilized on the chip to do the binding measurement. All the proteins were respectively immobilized on a CM5 sensor chip by using standard amine-coupling at 25°C with running buffer HEPES-P (20 mM HEPES buffer, 2.7 mM NaCl, 137 mM KCl, 0.05% surfactant P20, pH 7.4). A reference channel was only activated and blocked to eliminate compound unspecific binding to the surface of the chip. The immobilization level of EGFR extracellular ligand binding domain, EGFR intracellular kinase domain and GST were all about 10,000 RU. Different concentrations of compounds containing 5% DMSO were serially injected into the channel to evaluate the binding affinity. Extra wash with 50% DMSO was added to remove the last remaining sample in the pipeline. The association constants (k_a_), the dissociation constants (k_d_) and the equilibrium dissociation constants (K_D_) of the compounds were obtained by fitting the data sets to 1:1 Langmuir binding model using Biacore 8K Evaluation Software.

### 2.9 SPR based competition experiments

To elucidate the binding sites of the compounds exhibiting the highest affinity towards the extracellular ligand-binding domain and intracellular kinase domain of EGFR, Trichoharzin and Tandyukisin were selected based on their optimal binding affinities for competition experiments with EGF and ATP, respectively.

In the EGF competition experiments, a buffer scouting strategy using the A-B-A mode from the Biacore 8K method wizard was employed to assess the impact of EGF on the binding affinity of Trichoharzin and *vice versa*, towards the EGFR extracellular ligand-binding domain. The protocol fixed the pre-flank contact time, analyte contact time, and post-flank contact time at 60 s, 60 s, and 120 s, respectively. To evaluate the influence of EGF on Trichoharzin, A represented a single concentration of EGF, whereas B denoted a combination of varying concentrations of Trichoharzin with the identical EGF concentration as in A. Conversely, to examine the effect of Trichoharzin on EGF, A indicated a single concentration of Trichoharzin, and B comprised various EGF concentrations mixed with the same concentration of Trichoharzin as in A.

For ATP competition experiments, the LWM kinetic/affinity analysis protocol from the Biacore 8K method wizard was utilized to determine the effect of ATP on the binding affinity of compounds to the EGFR intracellular kinase domain. The investigation involved running buffers both with and without 1 mM ATP to ascertain whether ATP and the compounds compete for the same binding site.

### 2.10 Molecular docking and molecular dynamics simulation

The program Glide Standard Precise (SP) mode and Extra Precise (XP) mode with default parameters were proposed to do the molecular docking studies. The crystal structure of EGFR intracellular kinase domain (PDB code: 5CAV), CDK4 (PDB code: 2W96), CDK6 (PDB code: 3NUP), mTOR (PDB code: 3OAW), and HER2 (PDB code: 5O4G) was used as the target receptor for the docking of the compound. Tandyukisin with best cancer cell inhibition activities was successively docked into the above functional binding sites. Binding mode from XP was used to analyze key interactions between molecules and the corresponding binding site.

The docking results from Glide XP was also used for the binding mode prediction of Trichoharzin in EGFR extracellular ligand binding domain (PDB code: 1IVO).

Molecular dynamics (MD) simulations of ligand-protein complexes were conducted using Desmond in Schrodinger. Initially, a water model was established utilizing the System Builder tool, followed by the addition of sodium and chloride ions to achieve neutralization. The minimization processes employed the OPLS3 force field. Subsequently, the complexes underwent simulations for 1 ns and an extended 100 ns under NPT conditions at a temperature of 300 K. Trajectory analysis facilitated the extraction of key parameters, including the root mean square deviation (RMSD), root mean square fluctuation (RMSF), ligand contact maps, and binding profiles from the simulation data.

### 2.11 Synergistic experiments between Tandyukisin and Trichoharzin in enzymatic and cell-based assays

MCF-7 cells were seeded at a density of 5,000 cells per well in 96-well plates and allowed to adhere overnight. Subsequently, the cells were treated with various concentrations of Trichoharzin (0, 1.56, 3.125, 6.25, 12.5, 25, 50 µM) and Tandyukisin (0, 1.56, 3.125, 6.25, 12.5, 25, 50 µM) for 24 h. The efficacy of these treatments was assessed using the MTT assay to determine the inhibitory effects of the compound combinations.

In the enzymatic assay examining the combination of Tandyukisin and Trichoharzin, Tandyukisin was used at various concentrations (0, 0.31, 0.63, 1.25, 2.5, 5, 10, 20 nM). Each concentration of Tandyukisin was then combined with differing concentrations of Trichoharzin (0, 0.31, 0.63, 1.25, 2.5, 5, 10, 20 nM). The resultant mixtures were pre-incubated with enzyme samples to evaluate their inhibitory effects on EGFR activity.

### 2.12 Statistical analysis

All experiments were repeated at least three times, and the data are presented as mean ± standard deviation (SD). The statistical analyses were performed using Origin. EC_50_ or IC_50_ values were obtained by fitting the data to a four-parameter Hill model of the graph of log dose against percentage cell viability or inhibition from at least three sets of experiments. Difference between two groups were analyzed by Student’s t test (two-sided) and significance was set at *p* < 0.05. The specific details about statistical methods are introduced in respective figure legends.

## 3 Results

### 3.1 Tandyukisin inhibits MCF-7 growth by promoting early-stage cell apoptosis

**FIGURE 1:**
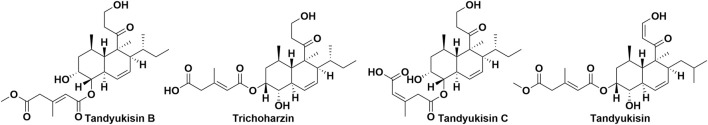
The chemical structures of Tandyukisin B, Trichoharzin, Tandyukisin C and Tandyukisin.

**FIGURE 2:**
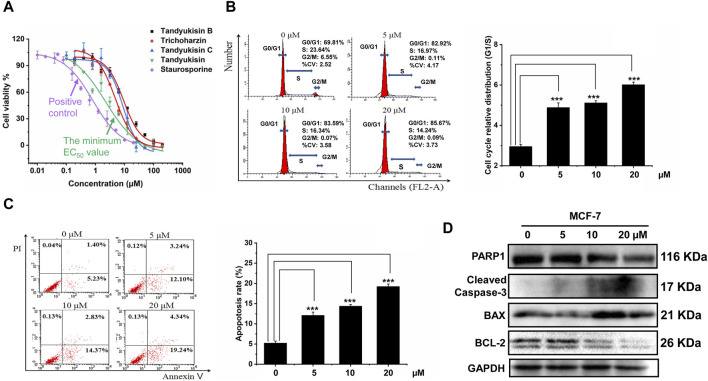
Identification of compounds that can significantly inhibit MCF-7 cell growth and induce cell apoptosis. **(A)** Growth inhibition activity of compounds in MCF-7 cells. The effect of treating MCF-7 cells with these increasing concentrations of compounds for 24 h was evaluated using MTT assay. **(B)** Percentage of MCF-7 cells in different phases of the cell cycle after respectively treatment with 5, 10, and 20 μM Tandyukisin for 24 h using flow cytometric analysis. **(C)** The effect of Tandyukisin in cell apoptosis of MCF-7 cells. The apoptosis of cells was confirmed by Annexin-V/PI double-staining after Tandyukisin treatment for 24 h. **(D)** The expression levels of PARP1 and cleaved caspase-3 in MCF-7 cells after Tandyukisin treatment. All values were repeated three times and represented as the mean ± SD. *p* values were obtained from a two-tailed Student’s t-test, *p < 0.05, **p < 0.01, ***p < 0.001.

According to data from the International Agency for Research on Cancer (IARC) released in December 2020, breast cancer has surpassed lung cancer as the most frequently diagnosed cancer worldwide. In light of this, our laboratory chose the MCF-7 breast cancer cell line for evaluating the bioactivity of 675 natural products. Initial screenings identified four compounds that significantly inhibited the proliferation of MCF-7 cells at a concentration of 10 μM, with their structures depicted in Figure 1. Subsequent dose-response analyses revealed that these compounds exerted dose-dependent inhibitory effects on cell viability at micromolar concentrations, as illustrated in Figure 2A. The half-maximal effective concentration (EC_50_) values for Tandyukisin B, Trichoharzin, Tandyukisin C, and Tandyukisin were determined to be 9.2 ± 1.2, 4.8 ± 0.4, 8.7 ± 1.0, and 3.0 ± 1.0 µM, respectively. The EC_50_ value for the positive control Staurosporine was 0.5 ± 0.2 µM.

To further assess the anti-proliferative properties of Tandyukisin, with the best cell growth inhibition activity, we analyzed its impact on the cell cycle of MCF-7 cells. Following 24 h of treatment at varying concentrations, the cells were collected for cell cycle analysis using flow cytometry. The results indicated a marked increase in the proportion of cells in the G0/G1 phase and a decrease in the S and G2/M phases at a concentration of 20 μM, elevating the G0/G1-phase cells to 85.67%, as shown in Figure 2B.

Additionally, the potential of Tandyukisin to induce apoptosis in MCF-7 cells was investigated employing the Annexin V-FITC/PI Apoptosis Detection Kit and immunoblotting techniques. Cells in early apoptosis were identified as PI negative and annexin V-FITC positive, whereas late apoptotic cells were positive for both markers. The data, presented in Figure 2C, revealed a significant increase in the percentage of apoptotic cells following Tandyukisin treatment, in a concentration-dependent manner. Specifically, apoptosis ratios in untreated MCF-7 cells were at 5.23%, while treatment with Tandyukisin at concentrations of 5, 10, and 20 µM resulted in apoptosis ratios of 12.10%, 14.37%, and 19.24%, respectively. Meanwhile, upon Tandyukisin treatment, the expression level of PARP1 was decreased, while the expression level of cleaved caspase-3 protein increased (Figure 2D; Supplementary Table S1).

These findings suggest that the antitumor activity of Tandyukisin may be attributed to its ability to modulate the cell cycle and induce early apoptosis through the activation of caspase-3.

### 3.2 Compound functions by targeting to EGFR

To further investigate which target compounds bound in, based on MCF-7 is the breast cancer cell line, we selected five widely recognized breast cancer treatment targets and docked Tandyukisin with best cancer cell growth inhibition activities to their respectively functional binding site. The five targets are mammalian target of rapamycin (mTOR) (Yu et al., 2001), human epidermal growth factor receptor 2 (HER2, ERBB2/neu) (Jerusalem et al., 2019), cyclin-dependent kinase 4/6 (CDK4/6) (Pandey et al., 2019), breast cancer susceptibility gene (BRCA) (Engel and Fischer, 2015), and EGFR (HER1/ERBB1) (Kjaer et al., 2018). The docking results, summarized in Supplementary Table S2, revealed that Tandyukisin displayed the highest binding affinity to the EGFR intracellular kinase active site, with an optimal docking score of −7.2 kcal/mol. Notably, the docking scores for Tandyukisin with CDK4 and CDK6 were −6.0 and −5.9 kcal/mol, respectively. No binding information of the other three proteins were obtained.

**FIGURE 3:**
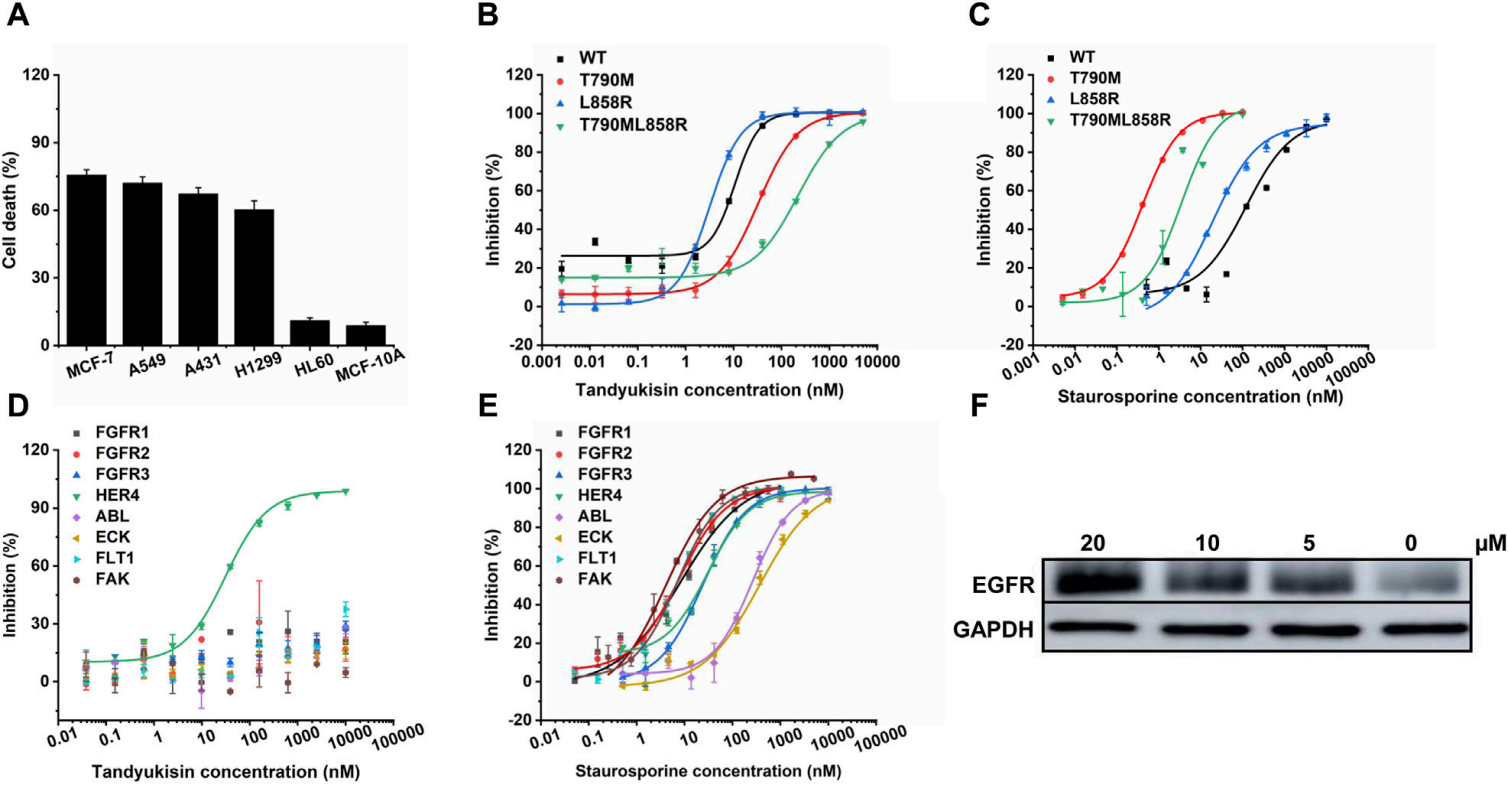
Compounds selectively target EGFR. **(A)** MTT assays of compound Tandyukisin for MCF-7, A549, A431, H1299, HL-60 and MCF-10A cells. **(B)** Inhibition of Tandyukisin on EGFR kinases determined by ELISA assay. **(C)** Inhibition of Staurosporine on EGFR kinases determined by ELISA assay. **(D)** Kinase profiling of Tandyukisin against eight human tyrosine kinases. The IC_50_ value of Tandyukisin for HER4 was 31.1 ± 0.1 nM, while the IC_50_ value of Tandyukisin for other seven tyrosine kinases were larger than 100 μM. **(E)** Kinase profiling of Staurosporine against eight human tyrosine kinases. The IC_50_ value of Staurosporine for FGFR1, FGFR2, FGFR3, HER4, ABL, ECK, FLT1 and FAK were 8.3 ± 2.0, 7.7 ± 0.9, 23.9 ± 1.0, 30.5 ± 0.1 nM, 262 ± 19, 361 ± 90, 7.0 ± 0.6 and 4.0 ± 2.0 nM, respectively. **(F)** CETSA was performed to evaluate the binding effects of Tandyukisin on the thermal stability of EGFR. IC_50_ values are shown as the mean ± SD from at least three independent experiments. Staurosporine was the positive control. FGFR1/2/3: fibroblast growth factor receptor 1/2/3, HER4: epidermal growth factor receptor 4, ABL: Abelson tyrosine-protein kinase, ECK: Epithelial cell kinase, FLT1: Vascular endothelial growth factor receptor 1, FAK: Focal Adhesion Kinase.

To further evaluate the selectivity and cytotoxicity of Tandyukisin across a range of cancer cell lines, we expanded our analysis beyond the EGFR-amplified breast cancer cell line MCF-7 to include high EGFR-expressing cancer cells (A549, A431, H1299) and low EGFR-expressing cancer cells (HL-60), as well as the non-tumorigenic epithelial cell line MCF-10A. The cytotoxic effects of Tandyukisin, at a concentration of 10 μM, were quantitatively assessed. The results, depicted in Figure 3A, indicated cell lethality percentages of 75.7% ± 2.3% for MCF-7, 72.1% ± 1.8% for A549, 67.3% ± 2.7% for A431, 60.3% ± 3.9% for H1299, 11.1% ± 1.2% for HL-60, and 8.9% ± 1.5% for MCF-10A, respectively.

The results demonstrate that the anti-tumor activity of Tandyukisin is largely achieved by targeting EGFR.

### 3.3 Tandyukisin targets both wild-type EGFR and its pathogenic mutants

In light of the significant challenge posed by drug-resistant mutations in EGFR-associated diseases, where over 50% of patients develop resistance, our research extended to evaluate the efficacy of compounds against both wild-type (WT) EGFR and prevalent drug-resistant mutants. Considering that the T790M point mutation in exon 20 confers resistance to EGFR-targeted therapeutics, and approximately two-thirds of patients treated with such drugs develop this mutation, its significance is underscored (Pao et al., 2005). Concurrently, the exon 19 deletion mutation (Ex19Del) and the exon 21 L858R point mutation represent the predominant pathogenic EGFR mutations, affecting the principal cohort treated with oral EGFR inhibitors, and constitute over 80% of all EGFR mutations (Rosell et al., 2009). Therefore, in our study on the enzyme activity of EGFR pathogenic mutants, we prioritized the inclusion of the T790M, L858R, and potentially the combined L858R/T790M mutations to align with their clinical prevalence and therapeutic relevance (Figure 3B, C; Supplementary Figure S1). The *in vitro* kinase assays demonstrated that the four studied compounds exhibited potent inhibition across these variants at nanomolar concentrations. Specifically, Tandyukisin showcased notable inhibitory concentrations (IC_50_) against WT EGFR, T790M, L858R, and L858R/T790M mutants, with values of 10.6 ± 0.6, 32.6 ± 0.6, 3.1 ± 0.1, and 237 ± 28 nM, respectively (Figure 3B). The IC_50_ values of positive control Staurosporine to WT, T790M, L858R, and L858R/T790M EGFR were 119.1 ± 0.1, 0.4 ± 0.1, 19.3 ± 0.1, and 3.4 ± 0.1 nM, respectively (Figure 3C). Remarkably, the IC_50_ for Tandyukisin against WT EGFR was determined to be 10 nM, revealing a potency 20-fold greater than that of the positive control Staurosporine against the same target.

To further ascertain the specificity of Tandyukisin’s action, we subjected it to tests against a panel of eight distinct tyrosine kinases. The results from these experiments elucidated that Tandyukisin exhibited selective inhibitory activity predominantly towards tyrosine kinases within the EGFR family (Figures 3D, E). Certainly, a more comprehensive evaluation of the selectivity and safety of Tandyukisin necessitates the validation across a broader spectrum of kinase types.

### 3.4 Tandyukisin efficiently promotes concentration-induced EGFR stability in MCF-7 cells

To confirm Tandyukisin directly bound EGFR in cells, CETSA was conducted. Currently, CETSA is widely used in drug target discovery assay and is based on the thermal stability changes after drugs bind to their targets (Martinez Molina et al., 2013; Jafari et al., 2014). After treatment with Tandyukisin at 5, 10, 20 μM for 24 h, the multiple aliquots of the cell suspension were heated at 55°C. The heated cells were subsequently lysed and centrifuged to separate soluble fractions from precipitated denatured EGFR. After thermal denaturation, EGFR in the soluble fraction was examined by Western blotting. As shown in Figure 3F; Supplementary Table S3, compared with DMSO control, cells treated with Tandyukisin showed a markable increase in the accumulation of EGFR in the soluble fraction at the examined temperatures, indicating the binding of Tandyukisin stabilized EGFR. At the protein level, Tandyukisin could also enhance the thermal stability of EGFR in a concentration-dependent manner, as demonstrated at concentrations of 1.3, 2.5, 5, 10, and 20 μM (Supplementary Figure S2).

### 3.5 Compounds specifically bind to EGFR by disrupting EGF or ATP binding

**FIGURE 4:**
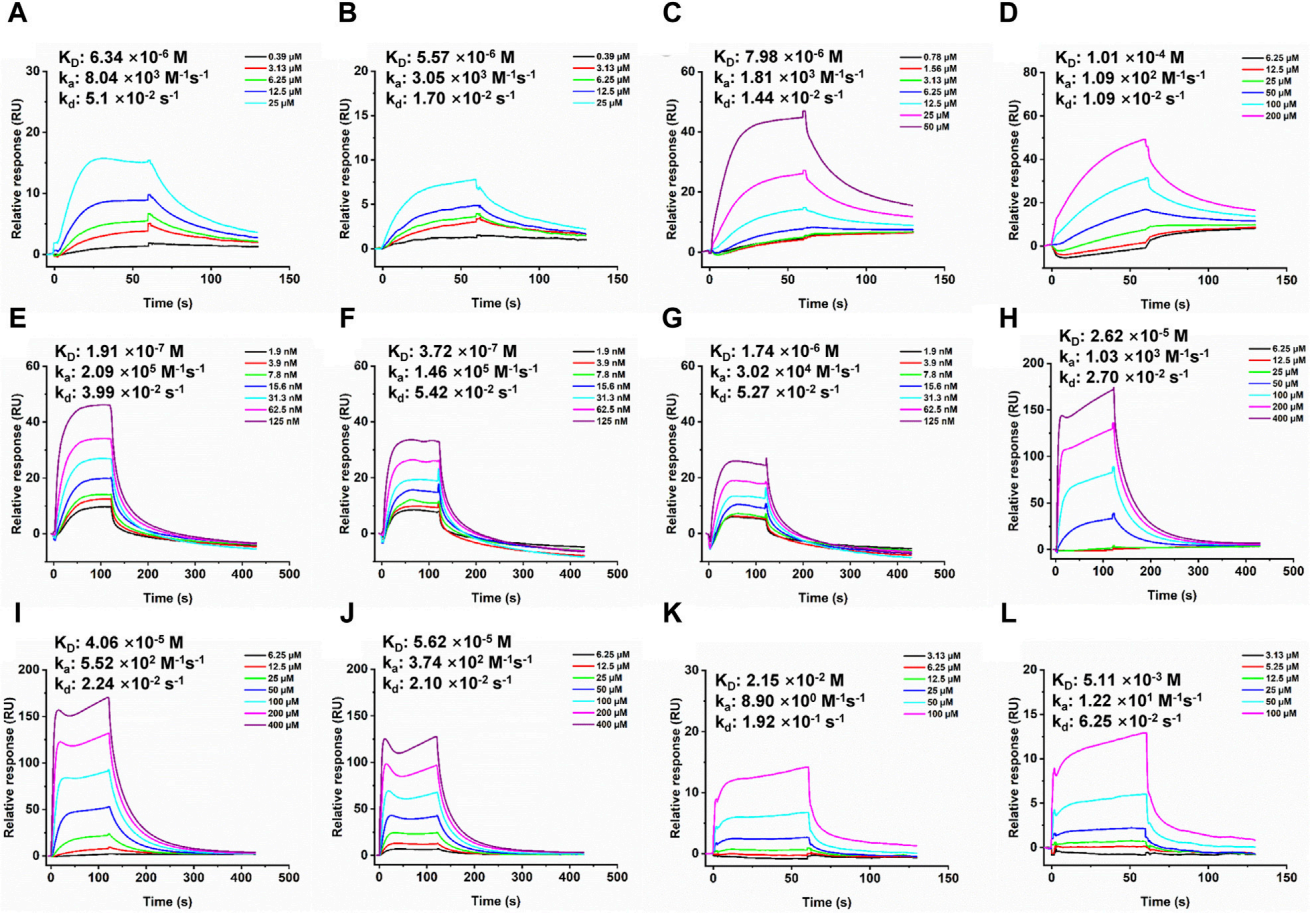
SPR dose response curves of Trichoharzin and Tandyukisin for EGFR. A-B. Trichoharzin **(A)** and Tandyukisin **(B)** bound to EGFR kinase domain without ATP. C-D. Trichoharzin **(C)** and Tandyukisin **(D)** bound to EGFR ligand binding domain. E-J. SPR competition experiment performed between Trichoharzin and EGF. In the presence of Trichoharzin at 125 **(E)**, 250 **(F)**, and 500 **(G)** µM, the K_D_ values of EGF decreased to 191, 372, and 1740 nM, respectively. **(** **H–J** **)**. In the presence of EGF at 2.5 **(H)**, 10 **(I)**, and 20 **(J)** nM, the K_D_ values of Trichoharzin reduced to 26.2, 40.6, and 56.2 µM, respectively. K-L. Trichoharzin **(K)** and Tandyukisin **(L)** bound to EGFR kinase domain in the presence of ATP.

To elucidate the direct interaction between EGFR and various compounds, SPR was employed. Given the membranous nature of EGFR, both its extracellular ligand-binding domain and intracellular kinase domain were procured for SPR analysis. An initial screening on a GST-immobilized chip was conducted to identify any nonspecific binding to the GST tag, which, fortunately, yielded no such interactions, as shown in Supplementary Figure S3. The sensorgrams suggested that the observed response changes were more attributable to bulk effects rather than specific interactions. All compounds showed concentration dependent binding responses for the two domains. The K_D_ values of compounds Tandyukisin B, Trichoharzin, Tandyukisin C and Tandyukisin for EGFR intracellular kinase domain were 7.02, 6.34, 6.56 and 5.57 µM, respectively (Figure 4A, B; Supplementary Figure S4A, B); while the K_D_ values of Tandyukisin B, Trichoharzin, Tandyukisin C and Tandyukisin for EGFR extracellular ligand binding domain were 30.0, 7.98, 55.1, and 101 μM, respectively Figure 4C, D; Supplementary Figure S4C, D).

The interaction between EGF and EGFR’s extracellular domain, known to trigger EGFR dimerization, autophosphorylation, and subsequent cellular proliferation and metastasis, was further explored (Citri and Yarden, 2006). A binding competition assay with Trichoharzin, which demonstrated the best affinity for the EGFR extracellular domain, was conducted to assess its potential to inhibit EGF-EGFR binding. SPR analysis confirmed a high-affinity interaction between EGF and EGFR (26.1 nM, Supplementary Figure S5), with Trichoharzin significantly inhibiting this interaction in a concentration-dependent manner, reducing the binding affinity of EGF to EGFR to 191, 372, and 1740 nM at concentrations of 125, 250, and 500 μM, respectively (Figures 4E–G). This represented a 7.3, 14.3, and 66.7-fold decrease in EGF binding affinity, respectively. A dose dependent decrease in the binding affinity for Trichoharzin upon addition of EGF at 0, 5, 10, and 20 nM was also observed. The K_D_ values of Trichoharzin against EGF at 2.5, 10, 20 nM incubated EGFR extracellular ligand binding domain were 26.2, 40.6, and 56.2 µM, respectively, which were also 3.3, 5.1, and 7.0 folds weaker than Trichoharzin binding alone (Figures 4H–J).

Considering EGFR belongs to receptor tyrosine family, we also examined the effect of ATP on compound binding by using EGFR intracellular kinase domain immobilized sensor chip. After adding 1 mM ATP to HEPES-P running buffer, nearly all compounds complete lost their binding ability to EGFR intracellular kinase domain and exhibited K_D_ values larger than 200 µM (Figure 4K, L; Supplementary Figure S6). This observation suggests that the presence of ATP significantly disrupts compound binding to the intracellular kinase domain of EGFR.

SPR results indicated that compounds can disrupt EGFR function by binding to EGF binding site, or ATP binding site, or both sites. SPR results also support that compounds did not aggregate under the experimental conditions.

### 3.6 Binding mode prediction

To elucidate the molecular interactions between the compounds and specific domains of EGFR, computational docking studies were performed using Glide (Friesner et al., 2004; Halgren et al., 2004), focusing on the binding of Trichoharzin to the EGF binding site and Tandyukisin to the ATP binding site.

The EGF/EGFR interaction interface is comprised of three primary sites: site I located in domain I, and sites II and III situated in domain III (Ogiso et al., 2002). At site I, the hydrophobic interactions involve the side chains of Leu14, Tyr45, Leu69, and Leu98 of EGFR with EGF, complemented by hydrogen bonding between residues 15–18 of EGFR and EGF. Site II is characterized by hydrophobic interactions involving Val350 and Phe357 of EGFR, a salt bridge formation by the Asp355 side chain with EGF, and van der Waals contacts between Phe357 and EGF. Site III features hydrophobic interactions with Leu382, Phe412, Val417, and Ile438 of EGFR, alongside a hydrogen bond formed by the Gln384 side chain with EGF.

**FIGURE 5:**
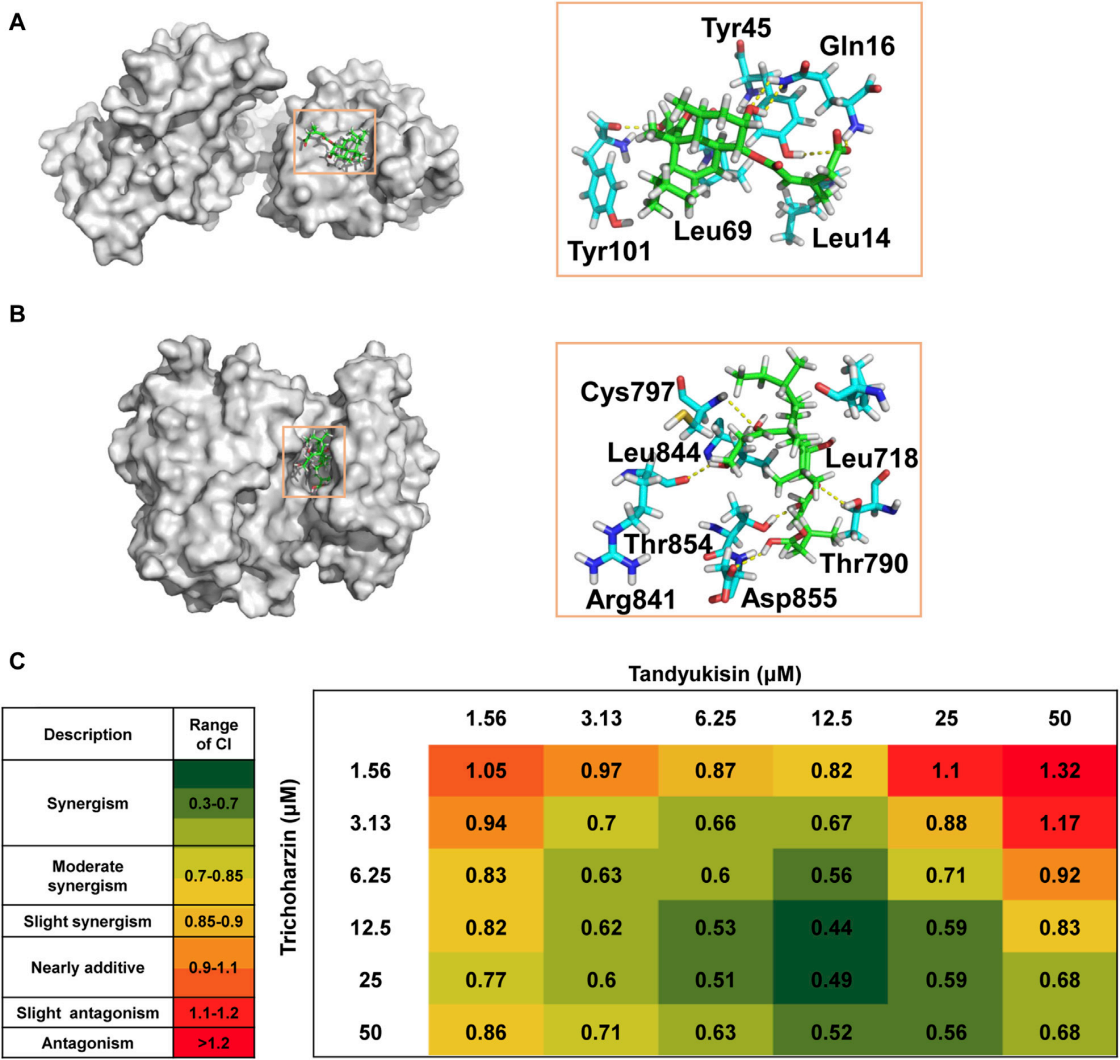
Trichoharzin and Tandyukisin synergize to induce rapid cell death in MCF-7 cell line. **(A)** The binding mode of Trichoharzin to EGFR ligand binding domain. **(B)** The binding mode of Tandyukisin to EGFR kinase domain. **(C)** CI values for each Trichoharzin and Tandyukisin combination were quantified by CompuSyn Software. CI values are heat mapped with lowest values in green and highest values in red.

Based on the docking results, Trichoharzin mainly occupied site I yielding a docking score of −6.2 kcal/mol (Figure 5A). Trichoharzin was anchored into site I by five hydrogen bonds, including a donor and two acceptor interaction with Gln16, an acceptor interaction with the polar side chain of Tyr45, and a donor interaction with the Tyr101 backbone. Hydrophobic interaction also exited between Trichoharzin and Leu 14 and Leu 69. Notably, the shallow nature of site I results in substantial exposure of Trichoharzin to the solvent.

The ATP binding site, a target for two generations of FDA-approved ATP-competitive inhibitors for cancer treatment, involves key residues such as Thr790, Met793, and Cys797 for drug-EGFR interactions (Hossam et al., 2016). The docking model of Tandyukisin highlighted seven EGFR residues in proximity (Figure 5B), with five crucial hydrogen-bond interactions involving donor and acceptor interactions with Cys797 and Arg841, an acceptor interaction with the Asp855 backbone, and donor interactions with the Thr854 and Thr790 backbones. Hydrophobic interactions are also observed between Tandyukisin and the alkyl side chains of Leu718 and Leu844. As illustrated in Supplementary Figure S7, MD simulation revealed that Tandyukisin could stably bind to the predicted sites within the kinetic simulation timeframe of 100 ns.

These docking results corroborate the experimental findings regarding the binding sites of the compounds, demonstrating a consistent alignment with the observed competition results. Considering that the pivotal residues facilitating the binding of Trichoharzin to EGFR are situated within the ligand-binding domain, and notwithstanding that the critical residues for the interaction of Tandyukisin with EGFR encompass Thr790, the hydrogen bond length formed by Tandyukisin and Thr790 measures 2.6 Angstroms. Consequently, the impact of this hydrogen bond on the interaction between Tandyukisin and EGFR appears non-decisive. This observation suggests a potential rationale for Trichoharzin and Tandyukisin exhibiting inhibitory activity against EGFR pathogenic mutants T790M and L858R.

### 3.7 Trichoharzin and Tandyukisin synergize to induce rapid cell death in MCF-7

Given the enhanced therapeutic efficacy, reduced side effects, and capability to circumvent multidrug resistance (MDR) associated with combination therapy, this approach has become a cornerstone in the treatment of cancer patients (Botham et al., 2014; Yang et al., 2015). In light of this, and following the identification of a suite of compounds exhibiting preferential binding to distinct sites on EGFR, we opted to investigate the combined effects of Trichoharzin, targeting the EGF binding site, and Tandyukisin, aimed at the ATP binding site, through MTT assays designed to assess their synergistic potential.

The synergy between Trichoharzin and Tandyukisin was quantitatively evaluated using the Chou and Talalay method (Chou, 2006), which calculates the Combination Index (CI). A CI value less than one signifies a synergistic interaction, a CI greater than one denotes antagonism, and a CI equal to one indicates an additive effect. Among these, the lowest CI value is indicative of the most potent synergistic interaction. The results of synergistic experiments for Trichoharzin and Tandyukisin in cell assays were shown in Figure 5C; Supplementary Figure S8. As can be seen, the combination of Trichoharzin and Tandyukisin creates a synergistic anti-tumor effect as CI values are lower than 1. The relative strong synergistic interactions (CI values between 0.4 and 0.6) were observed with Trichoharzin concentration ranging from 6.25 to 50 μM, while Tandyukisin concentration ranging from 6.25 to 25 µM. When the concentration of Trichoharzin is 1.56 µM or the concentration of Tandyukisin is 50 µM in the matrix, antagonistic interaction was more likely observed. Moreover, the synergistic effect of Trichoharzin and Tandyukisin on WT EGFR enzyme activity was investigated. The experimental results demonstrated a consistent synergistic effect almost across all concentrations of Trichoharzin within the concentration range of 1.25–10 nM of Tandyukisin, as depicted in Supplementary Figure S9. Synergistic results further confirm our competition and docking results about the binding sites of the compounds.

## 4 Discussion

From our in-house natural products library, we successfully identified a novel EGFR inhibitor, Tandyukisin, characterized by a unique chemical scaffold. Tandyukisin has demonstrated the ability to inhibit cancer cell growth in the micromolar range, selectively target EGFR-amplified breast cancer cell proliferation, and induce apoptosis through Caspase-3 activation. It effectively inhibited both wild-type and mutant forms of EGFR (T790M, L858R, and L858R/T790M) with a concentration range from 3 to 240 nM, showcasing pronounced selectivity for EGFR tyrosine kinase. The use of CETSA demonstrated that Tandyukisin targeted EGFR in cells and promoted concentration-induced EGFR stability. SPR assay provided the exact binding affinity of Tandyukisin to EGFR. Moreover, competition experiments between Tandyukisin or Trichoharzin and ATP or EGF further confirmed the truth that compound bound in the orthogonal site. Molecular docking studies specified Cys797, Arg 841, Asp855, Thr854 and Thr790 were the key residues to stabilize Tandyukisin in the site. In addition, Tandyukisin and Trichoharzin used in combination can accelerate the death of breast cancer cells.

This marks the inaugural report of Tandyukisin as an EGFR inhibitor, although its anti-tumor properties were previously identified in a study by Takeshi Yamada’s group in 2015, which isolated a new decalin derivative from the marine sponge Halichondria okadai (Yamada et al., 2015). They characterized the structure of the compound using 1D and 2D NMR techniques, and the compounds were then tested for their liver cancer cell inhibitory activity. Marine organisms, thriving under extreme environmental conditions, are prolific producers of bioactive compounds with potential anti-tumor, antibacterial, and antiviral properties (Bull et al., 2005). According to incomplete statistics, more than 25,000 marine natural product compounds have been studied so far, and they are expected to be further developed into marine drugs and other marine biological products. There are currently about 14 marine drugs approved for marketing, and more than 50 marine drugs are in the clinical trial stage (Ruiz-Torres et al., 2017), underscoring the marine ecosystem’s vast potential for novel drug discovery, particularly in oncology. However, due to the relatively low distribution density of marine biological resources, and the anti-tumor active substances in marine organisms are at the microgram or milligram level, the large-scale preparation of active substances has become one of the keys limiting factors in the development of marine anti-tumor drugs.

Theoretically, the binding affinity of inhibitor (K_D_ value) in the orthogonal site should be coordinate with its enzymatic inhibition ability (IC_50_ value). For Tandyukisin, its K_D_ value is 5.57 µM, while its EGFR inhibition activity IC_50_ value is about 10 nM. The difference between K_D_ and IC_50_ value is mainly caused by that the compound may inhibit enzyme activity by inducing conformational changes in the kinase domain. Immobilizing the kinase domain on the chip will hinder the conformational changes of the protein, so it showed weaker binding activity. Another reason may be related to the inconsistent activity status of the protein under different experimental conditions. The mechanism of Tandyukisin inhibiting EGFR activity involves two main actions: firstly, by directly occupying the ATP binding site, and secondly, potentially by restricting the mobility of the kinase domain’s rigid regions. This restriction could prevent the active site from adopting a closed conformation, thereby stabilizing EGFR in an inactive state. However, elucidating the precise mechanism of inhibition necessitates further experimental and computational investigations to fully understand how Tandyukisin influences EGFR activity and conformation.

Although a small number of cancer patients can benefit from EGFR inhibitors as monotherapy in patients with malignancies such as breast cancer and non-small cell lung cancer, cancer cells may activate different mechanisms to evade the antitumor activity of EGFR-targeted anti-tumor drugs. Consequently, combination therapy involving EGFR inhibitors is increasingly favored due to its potential to surmount acquired resistance, thereby enhancing the responsiveness of drug-resistant cells and extending patient progression-free survival (PFS). As per predictions from ADMET lab3.0 (https://admetlab3.scbdd.com/), Tandyukisin demonstrates low toxicity across three commonly utilized cell lines (RPMI-8226, A549, HEK293) and exhibits minimal adverse effects on neurological, genetic, and cardiac functions, among others, indicating overall favorable performance. However, the conjugated structure of Tandyukisin’s active double-bonded carbonyl group may impact its distribution and metabolism, potentially leading to renal and respiratory toxicity. Nonetheless, Tandyukisin shows no inhibitory effects on most P450 isoenzymes in the human body and exhibits excellent stability in human liver microsomes (HLM), thereby not interfering with the pharmacokinetic properties of other therapeutic drugs. Hence, Tandyukisin presents itself as highly suitable for combination therapy. We anticipate that our discovery of an EGFR inhibitor with a novel scaffold will address the issue of low yield post-completion of full chemical synthesis steps. However, before Tandyukisin can progress to clinical trials, it necessitates pharmacophore optimization, scaffold transition, and other medicinal chemistry approaches to enhance its pharmacokinetic properties and optimize drug efficacy. The strategic co-administration of EGFR inhibitors has the potential to amplify therapeutic outcomes and mitigate the risk of resistance development. Of course, the safety and efficacy of such combination therapies necessitate further comprehensive investigation.

## 5 Conclusion

In this study, we have identified Tandyukisin as a novel EGFR inhibitor capable of significantly suppressing the enzymatic activity of both EGFR and its drug-resistant mutants at nanomolar concentrations. Derived from compounds isolated from marine organisms, Tandyukisin offers valuable insights for the development of marine-derived antitumor drugs. The EGFR receptor can activate three signaling pathways, namely, the JAK/STAT signaling pathway involved in immune regulation, the RAS-RAF-MEK pathway (MAPK/ERK pathway) involved in cell proliferation, and the PI3K-AKT-mTOR pathway involved in cell survival. Our research has demonstrated that EGFR inhibitors can activate Caspase-3, increase BAX, and reduce BCL-2 to promote early apoptosis of cells. However, further investigation into molecular and protein-level mechanisms, such as the impact of BCL-2 like protein 11 mRNA and protein expression, and the phosphorylation levels of EGFR, AKT, and ERK, should be pursued diligently. Despite the current limitations in the yield of Tandyukisin impeding further *in vitro* or *in vivo* experimental validation, the identification of Tandyukisin could facilitate the development of next-generation EGFR inhibitors and enable the investigation of their potential synergistic effects with inhibitors that target related pathways.

## Data Availability

The original contributions presented in the study are included in the article/Supplementary Material, further inquiries can be directed to the corresponding author.
