# Supplementary material for: Marine-derived EGFR inhibitors: novel compounds targeting breast cancer growth and drug resistance
Source: Front Pharmacol. 2024 May 1;15:1396605. doi: 10.3389/fphar.2024.1396605 (PMC11094307; doi:10.3389/fphar.2024.1396605)
Supplement: Supplementary file 1 [file DataSheet1.docx]

**Marine-Derived EGFR Inhibitors: Novel Compounds Targeting Breast Cancer Growth and Drug Resistance**

Qi Li^1,#^, Bo Li^1,#^, Qian Wang^1,#,*^, Chengen Wang^2^, Miao Yu^3^, Tianfu Xu^1^

^1^State Key Laboratory of Natural and Biomimetic Drugs, School of Pharmaceutical Sciences, Peking University, Beijing, 100191, China

^2^Department of Minimally Invasive Tumor Therapies Center, Beijing Hospital, National Center of Gerontology, Institute of Geriatric Medicine, Chinese Academy of Medical Sciences, No.1 Da Hua Road, Dong Dan, 100730 Beijing, China

^3^Engineering Research Center for Medicine, Ministry of Education, Harbin University of Commerce, Harbin, 150076, China

^#^These authors contributed equally to this work

^*^Corresponding author: Qian Wang, 010-82801437, qian.wang@bjmu.edu.cn

Tables

Table S1. The gray value of WB results from Figure 2D.

|  | **Concentration (µM)** | **Gray Value** | **Normalized** |
| --- | --- | --- | --- |
| **PAPR1** | 0 | 41093.288 | 1.034604877 |
|  | 5 | 43160.066 | 0.984358216 |
|  | 10 | 33044.409 | 0.758999438 |
|  | 20 | 24731.459 | 0.723158188 |
| **Cleaved Caspase-3** | 0 | 8469.459 | 0.213235397 |
|  | 5 | 17172.004 | 0.391644518 |
|  | 10 | 22734.418 | 0.522188503 |
|  | 20 | 173083.238 | 5.061026155 |
| **BAX** | 0 | 31579.501 | 0.795076455 |
|  | 5 | 19561.731 | 0.446147386 |
|  | 10 | 48768.744 | 1.120172834 |
|  | 20 | 33049.489 | 0.966380859 |
| **BCL-2** | 0 | 33435.995 | 0.841817367 |
|  | 5 | 41097.258 | 0.937311439 |
|  | 10 | 21588.48 | 0.49586737 |
|  | 20 | 7215.723 | 0.210990754 |
| **GAPDH** | 0 | 39718.823 |  |
|  | 5 | 43845.894 |  |
|  | 10 | 43536.803 |  |
|  | 20 | 34199.238 |  |

Table S2. Summary table of docking scores for Tandyukisin and breast cancer-related targets.

| **Target** | **PDB code** | **Docking score (Kcal/mol)** |
| --- | --- | --- |
| **EGFR intracellular kinase domain** | 5CAV | -7.2 |
| **CDK4** | 2W96 | -6.0 |
| **CDK6** | 3NUP | -5.0 |
| **HER2** | 5O4G | > -4.0 |
| **BRCA** | No suitable docking structure | ^a^/ |
| **mTOR** | 3OAW | > -4.0 |

^a^ Not determined.

Table S3. The gray value of WB results from Figure 3F.

|  | **Concentration (µM)** | **Gray Value** | **Normalized** |
| --- | --- | --- | --- |
| **PAPR1** | 20 | 42106.53 | 1.165872275 |
|  | 10 | 31150.702 | 0.806312599 |
|  | 5 | 29078.924 | 0.70192391 |
|  | 0 | 21019.095 | 0.614197892 |
| **GAPDH** | 20 | 36115.903 |  |
|  | 10 | 38633.53 |  |
|  | 5 | 41427.459 |  |
|  | 0 | 34222.024 |  |

Figures


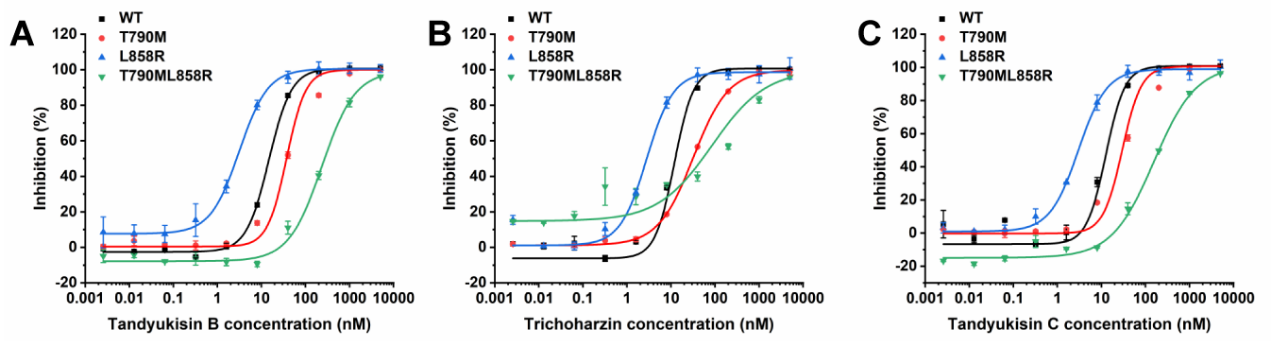


Figure S1. Enzyme activity inhibition curves of Tandyukisin B, Trichoharzin, and Tandyukisin C against WT EGFR and EGFR pathogenic mutants. IC_50_ values of Tandyukisin B (A) against WT, T790M, L858R, and L858R/T790M were 14.7 ± 1.1, 37.3 ± 0.1, 3.1 ± 0.1, and 237 ± 28 nM, respectively. IC_50_ values of Trichoharzin (B) against WT, T790M, L858R, and L858R/T790M were 11.9 ± 0.1, 32.0 ± 0.5, 2.8 ± 0.6, and 85.5 ± 27.7 nM, respectively. IC_50_ values of Tandyukisin C (C) against WT, T790M, L858R, and L858R/T790M were 12.6 ± 0.1, 29.7 ± 0.1, 3.0 ± 0.2, and 156 ± 4 nM, respectively.


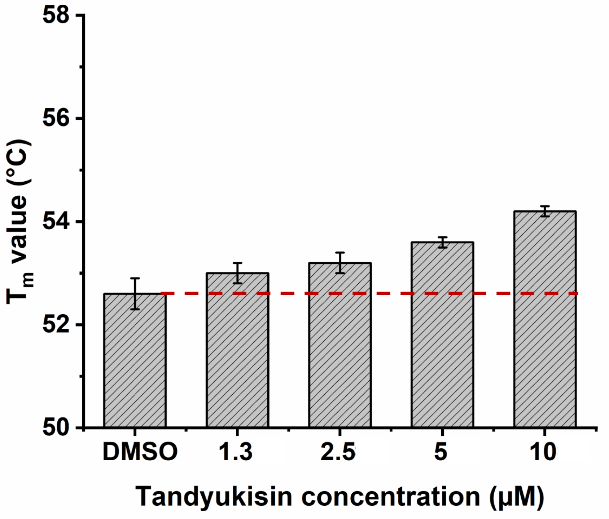


Figure S2. The impact of Tandyukisin on the thermal stability of the EGFR kinase domain. As the concentration of Tandyukisin increased from 0 to 10 µM, there was a corresponding increase in the melting temperature (T_m_) of EGFR from 52.6°C to 54.2°C, indicating enhanced thermal stability.


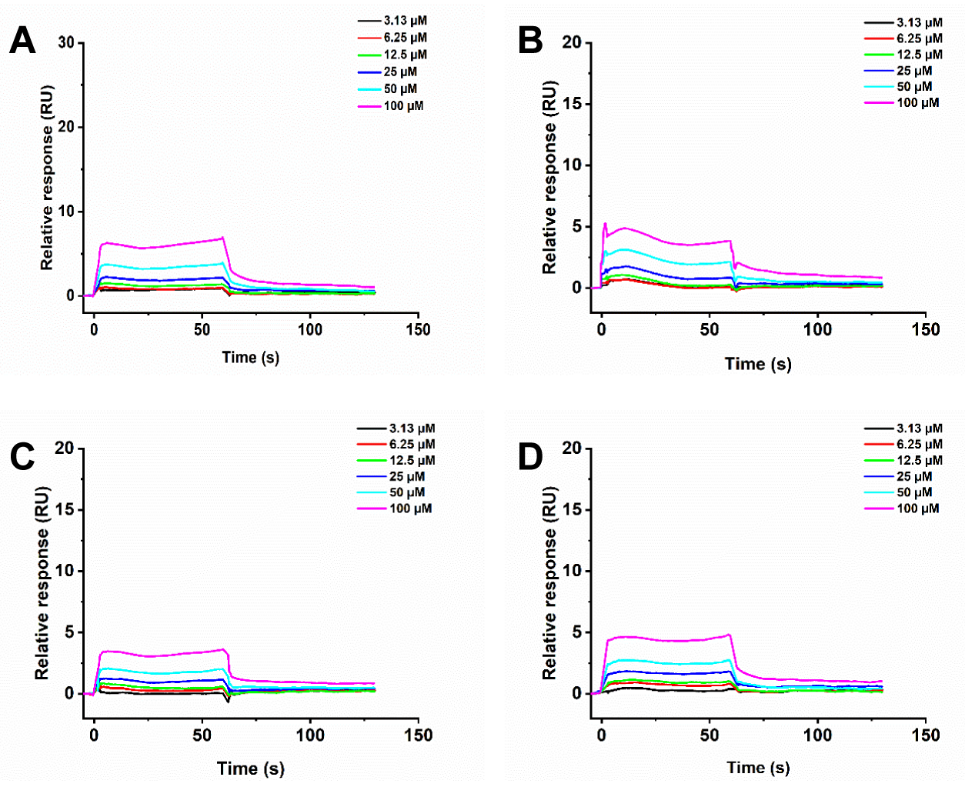


Figure S3. SPR sensorgram of Tandyukisin B, Trichoharzin, Tandyukisin C and Tandyukisin binding to GST protein. The SPR association and dissociation curves exhibited a fast on and off binding mode, and did not reach a steady state.


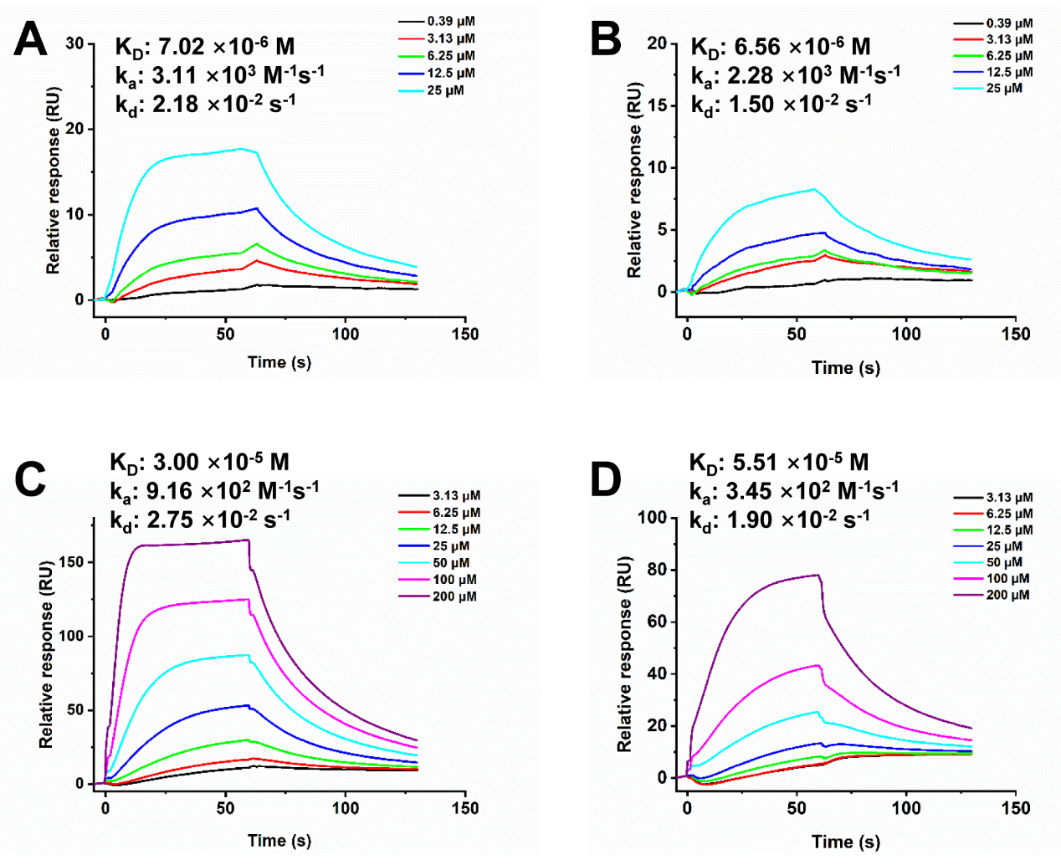


Figure S4. A-B. SPR sensorgram of Tandyukisin B (A) and Tandyukisin C (B) binding to the intracellular kinase domain of EGFR in the absence of ATP. C-D. SPR sensorgrams of Tandyukisin B and Tandyukisin C binding to the extracellular ligand binding domain of EGFR.


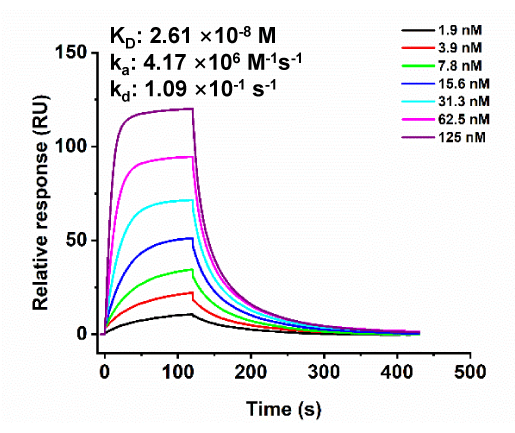


Figure S5. SPR results of EGF and EGFR ligand binding domains.


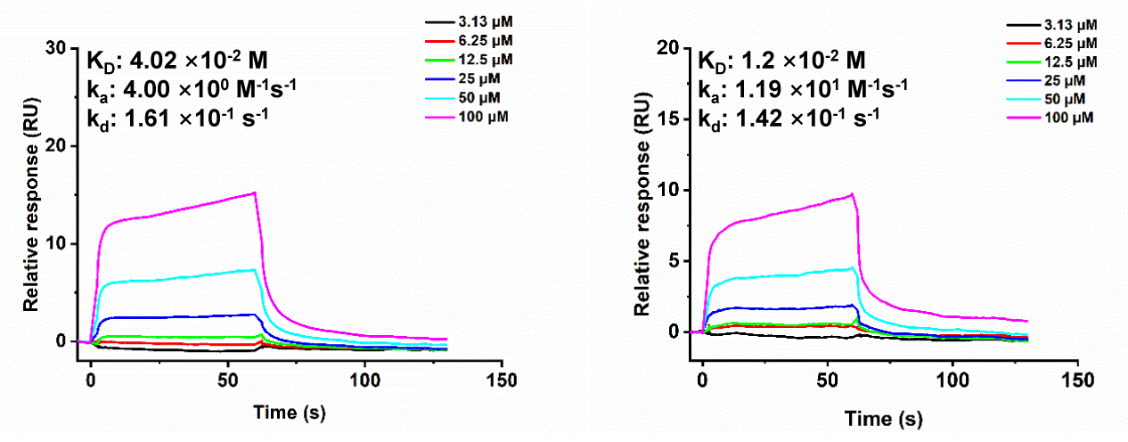


Figure S6. In the presence of ATP, Tandyukisin B (left) and Tandyukisin C (right) hardly bind to the extracellular kinase domain of EGFR.





Figure S7. The MD results of Tandyulisin for EGFR kinase domain.


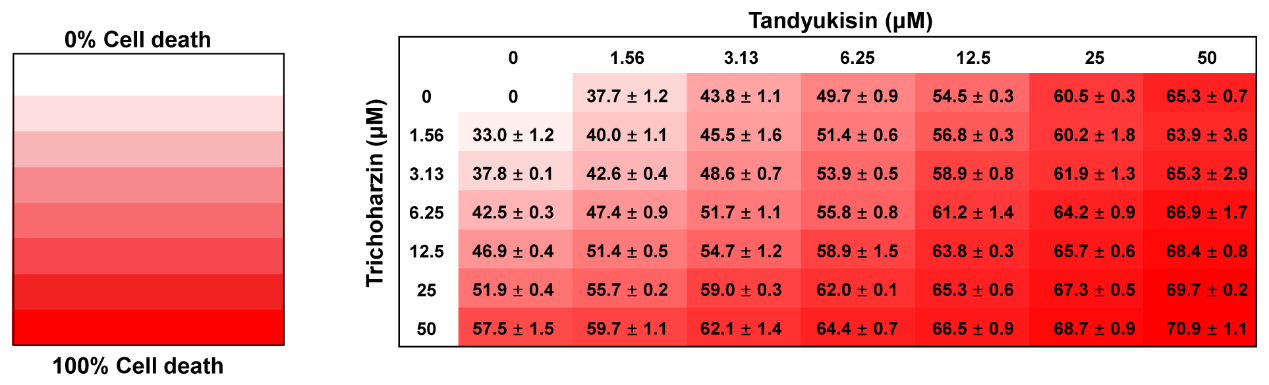


Figure S8. Percent cell death observed after 24 h treatment of Tandyukisin and Trichoharzin in matrix format.


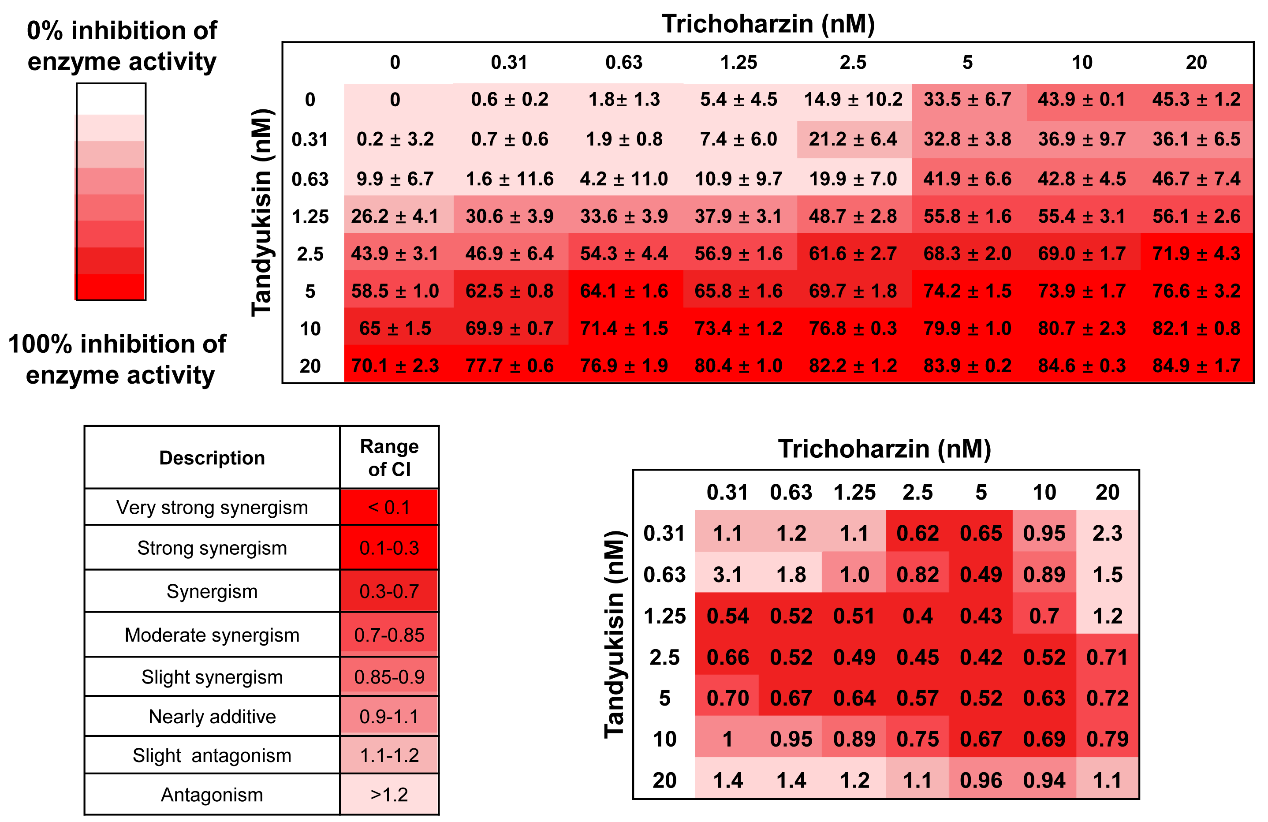


Figure S9. The synergism of Tandyukisin and Trichoharzin in the enzymatic assay.
